# Role of Magnesium Supplementation in Children with West Syndrome: A Randomized Controlled Clinical Trial

**DOI:** 10.22037/ijcn.v16i1.30480

**Published:** 2022-01-01

**Authors:** Vijay Kumar YADAV, Amrita AMRITA, Sunita YADAV, Rajeev KUMAR, Krishna Kumar YADAV

**Affiliations:** 1 Department of Pediatrics, M.R.A. Medical College, Ambedkar Nagar, UP, India.; 2 Department of Pathology, M.R.A. Medical College, Ambedkarnagar UP India.; 3 Rajeev Kumar, DNB, SR Pediatric Critical Care, Narayana Hrudayalaya, Bengaluru, India; 4 R.M.L. Institute of Medical Sciences, Lucknow, India.

**Keywords:** Magnesium supplementation, West syndrome, Seizures

## Abstract

**Objectives:**

West syndrome is a severe epileptic encephalopathy of young age. It is characterized by a clinico-electrical triad of infantile epileptic spasms, regression or arrest of psychomotor development, and hypsarrhythmia. In the last two decades, the large progress in the development of newer antiepileptic drugs has allowed us to have a vast choice of treatment options to control spasms, although they often fail to do so. Thus, there is a need to explore other treatment options.

**Materials & Methods:**

Subjects in this open-labelled randomized control trial were included newly diagnosed children of age between 3 months and 5 years of both genders. A total of 52 children were recruited and randomized into two groups: an intervention group (n=30) and a non-intervention group (n=22). Magnesium sulphate was provided for the intervention group but not for the non-intervention one. Both groups received the rest of the treatments, including adrenocorticotropic hormone and antiepileptic drugs. The follow-up period was three months, at the end of which a per-protocol analysis was performed.

**Results:**

There was no significant difference in seizure control and neurodevelopmental outcome between both groups, but electroencephalogram significantly improved in the intervention group compared to the control. Also, the clinical response was better in patients with normal initial serum magnesium levels in the intervention group (p=0.003) than in other patients.

**Conclusion:**

Magnesium supplementation may be helpful in children with West syndrome.

## Introduction

West syndrome (WS) is a severe epileptic encephalopathy of young age. It is characterized by a clinico-electrical triad of (i) infantile epileptic spasms, (ii) regression or arrest of psychomotor development, and (iii) hypsarrhythmia (1). Hypsarrhythmia is a slow, chaotic background with multifocal spikes of high voltage on electroencephalography (EEG) (2). This classical triad comprises variations in age of onset ranging from one month to 4 years: spasms that may be single, asymmetrical, or combined with focal seizures; asymmetrical, synchronous, or fragmented hypsarrhythmia; and psychomotor function that may be delayed, deteriorated, or normal (1). WS is one of the most typical infantile epileptic syndromes seen in pediatric neurology clinics with an age of onset mostly between 3-12 months, peaking around 5 months of age with a male preponderance (3). 

There is no agreement on the optimum treatment for WS. In the last two decades, the large progress in the development of newer antiepileptic drugs has enabled us to have a vast choice of treatment options to control spasms, although they often fail to do so (4,5). Thus, there is a need to explore other treatment options.

Various studies have shown lower serum levels of magnesium in children with epilepsy (6-13). Magnesium has antiepileptic and neuroprotective properties due to the antagonizing effect of the N-methyl-d–aspartate (NMDA) receptor (14). Magnesium is useful in various refractory epilepsies (15-17). However, there is a dearth of studies, especially randomized control trials (RCTs), on the role of magnesium in children with WS. Therefore, the present study aimed to examine the effect of magnesium on children with WS regarding seizure control, neurodevelopmental outcome, and EEG improvement through an RCT study design.

## Materials & Methods

This open-labeled randomized controlled clinical trial was conducted at the pediatrics department of a government tertiary care teaching hospital of Northern India from Feb 2019 to March 2020 in children with WS aged between 3 months and 5 years. Subjects included were newly diagnosed cases of WS having two or more of these three criteria: infantile epileptic spasms, hypsarrhythmia on EEG, and psychomotor retardation/regression. Old and previously diagnosed cases of WS, acutely sick children, children aged below 3 months and above 5 years, and children with cardiac diseases or renal failure were excluded from the study. Patients without signed consent forms or those provided with prior magnesium supplementation within 3 months were also excluded. Informed written consent for inclusion in the study was obtained from the patients’ parents or guardians after explaining the procedures and involved risks and benefits to them. Ethical clearance was obtained from the Ethical Committee of the Institute. 

Detailed clinical history, including patients particulars, details of infantile spasms, perinatal and neonatal history, developmental history, thorough clinical examination, including general-physical examinations, anthropometric measurement, and detailed central nervous system examination, fundus and vision (if required), and follow-up were recorded in a predesigned proforma. 

During the follow-up, proforma was updated on every visit/interaction with the parents. A 16 channel sleep EEG was done and neuroimaging in the form of magnetic resonance imaging of the brain or cranial computerized tomographic scan was carried out at the department of radiodiagnosis. Also, development quotient/social quotient (DQ/SQ) assessment were done at the department of psychiatry using standard scales, including the developmental screening test (DST)/the Vineland social maturity scale (VSMS). Serum magnesium level estimation was carried out in the laboratory of the department of biochemistry using the Colorimetric Xylidyl Blue method, and the serum magnesium level less than 1.5 mg% was considered low. 

The sample size was calculated based on Zou LP et al.’s study (2010) (18). Accordingly, the recurrence of seizures in patients with WS is reduced by 75% with magnesium supplementation and by 50% and type 1 error of 10% (two-tailed) without magnesium supplementation. Thus, 25 patients in each group were required.

All the enrolled children were randomized into an intervention group and a non-intervention group using a computer-generated random number table. At enrolment, the intervention group received daily magnesium sulfate intramuscular injection for 3 consecutive days at recommended doses of 100mg/kg/day once a day (magnesium sulfate injection 50% w/v) followed by oral magnesium supplementation (children <6 months, 37.5mg/day and children  6 months, 75 mg/day) for 3 months as a syrup containing magnesium, calcium, and vitamin D. However, the non-intervention group received syrup containing an equal amount of calcium and vitamin D but without magnesium (age <6 months: calcium 100 mg/ day + vitamin D 100 IU/day; age   6 months – calcium 200 mg/day + vitamin D 200 IU/day). Supplementation was stopped in children with side effects due to magnesium. Both groups received injection adrenocorticotropin hormone (ACTH) and antiepileptic drugs according to a fixed protocol. 

All the children were followed up in detail at least once in 2 weeks up to 3 months of starting the treatment. The patients were assessed for seizure control and clinical improvement, and the proforma was filled at every visit. After 3 months, EEG and DQ/SQ were repeated, and the response was assessed in the form of EEG improvement, improvement in DQ/SQ levels, and the clinical response to the treatment. The improvements were categorized into three groups: (i) no response with <25% frequency reduction in seizures after 3 months of starting the treatment; (ii) partial response with >25% frequency reduction in spasms without the occurrence of other types of seizures, and (iii) complete response with the total control of seizures after 3 months of starting the treatment. 

The data was entered in an MS-Excel worksheet and analysed using the Epi info software. The mean of frequencies and standard deviations were calculated for descriptive data. Also, the chi-square test was used for categorical data, and the Student’s t-test was used for continuous data. Moreover, the one-way ANOVA test was used to compare serum magnesium levels and clinical responses to the treatment. A p-value of less than 0.05 was considered significant. 

## Results

During the study period, 52 patients were recruited, and their clinical profiles and demographics were studied. The intervention group included 30 patients, and the non-intervention group contained 22 patients. Two of the patients were lost during the follow-up, and two of the patients were excluded due to side effects from magnesium supplementation. Thus, at the end of the 3-month follow-up, 48 patients (26 patients in the intervention group and 22 patients in the non-intervention group) remained who were followed up and analysed in detail. 

We observed in our cohort that males were predominant with the male to female ratio of 3:1. The most common etiological factor was a perinatal insult. Most of the patients belonged to middle and low socioeconomic status. Most of the patients (63.5%) had flexor type of spasms occurring in series, and about one-third of them (36.5%) had seizures other than infantile spasms, with the most typical type being a generalized tonic-clonic seizure. Moreover, about half of the patients had microcephaly (48.1%), and about sixty percent of children with WS were undernutrition. Additionally, most of the patients had cerebral palsy, with spastic quadriplegia being the most typical type. 

The mean age of onset of spasms was 7.25 ± 6.69 months, and most of the patients (84.6%) had onset of spasms before 12 months of age. Patients with WS had DQ on the lower side, with the mean being 38.65 ± 15.97. Both groups were comparable regarding basic clinical profiles like sex and socioeconomic. Serum magnesium levels were low in 17 of the patients (35.41%), and the mean serum magnesium level was 2.07 ± 0.5 mg/dl. 

We found no significant difference between the groups after magnesium supplementation regarding clinical response/seizure control (p=0.69) and neuro-developmental outcome (p=0.09) but observed a significant difference in EEG improvement between the groups (p=0.04). We also observed that the clinical response was better in the intervention group when they had normal initial serum magnesium levels compared to low initial serum magnesium levels (p=0.003). However, we detected no significant difference in the non-intervention group regarding the clinical response at different serum magnesium levels (p=0.418). We also observed that patients with normal initial serum magnesium levels had complete responses compared to patients with low initial serum magnesium levels (p=0.004), meaning higher serum magnesium levels yielded a better clinical response. We detected no statistically significant difference in the response rate between the groups with normal initial serum magnesium levels. 


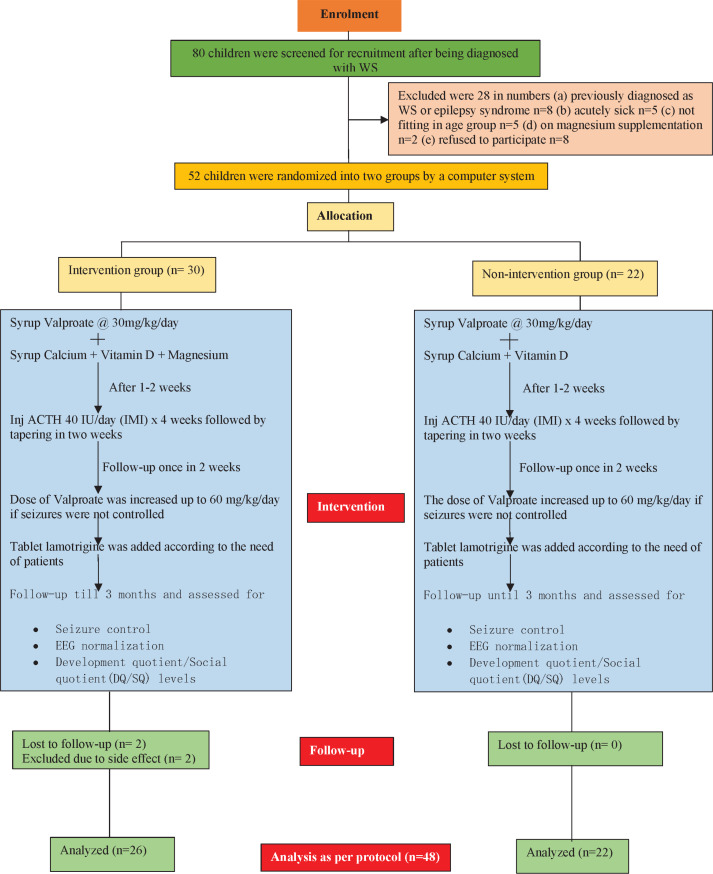


Figure 1: The flow of the study

**Table 1:** Baseline characteristics of both groups at the time of recruitment

S. No.	Characteristics		Intervention group (n=26)	Non-intervention Group (n=22)	p-value
1.	Sex	Male	21	15	0.32
		Female	5	7	
2.	Socio-economic status	Middle class	19	14	0.48
		Lower class	7	8	
3.	Nutritional status	Normal	8	11	0.38
		Moderate UN	6	3	
		Severe UN	12	8	
4.	Serum Magnesium levels	Normal	18	13	0.46
		Low	8	9	
5.	Serum Magnesium levels (mg/dl) Mean ± SD		2.10 ± 0.52	2.04 ± 0.55	0.71
6.	Frequency of spasms/day	< 5	11	5	0.32
		5-10	10	10	
		>10	5	7	
7.	Number of spasms per episode	Single	3	7	0.09
		Series	23	17	
8.	Seizures other than Infantile Spasms	Present	11	12	0.31
		Absent	15	10	
9.	Development Quotient	< 30	8	7	0.97
		30-60	15	12	
> 60	3	3

Abbreviations: UN- undernutrition

**Table 2:** Outcomes after 3 months of magnesium supplementation

S/No.	Characteristics		Intervention Group (n=26)	Non- intervention group (n=22)	p value
1.	Clinical Response	No	6	7	0.69
		Partial	7	4	
		Complete	13	11	
2.	Final EEG in initial abnormal EEG (n=26)	Normal	11	6	0.04
		Abnormal	2	7	
3.	DQ/SQ	Normal	4	0	0.09
Abnormal	22	22

Abbreviations: DQ- development quotient; SQ- social quotient

**Table 3:** Comparison of clinical response after magnesium supplementation with initial serum levels of magnesium in both groups

Category	Serum Magnesium level	Response to treatment			Total	p-value
		No	Partial	Complete		
Intervention group (n=26)	Normal	2	3	13	18	0.003
	Low	4	4	0	8	
Non-intervention group (n=22)	Normal	3	2	8	13	0.42
Low	4	2	3	9

**Table 4:** Comparison of Clinical response at 3 months with initial serum levels of magnesium

Serum Magnesium levels	Seizure control			p-value
	No response	Partial response	Complete response	
Normal	5	5	21	0.004
Low	8	6	3	
Total	13	11	24	

## Discussion

WS is an age-specific severe epileptic encephalopathy. It is characterized by a classical clinico-electrical triad, including infantile epileptic spasms, regression/arrest of psychomotor development, and hypsarrhythmia on EEG, although one element may be missing (1). Although WS is a well-known syndrome, its treatment is often unsatisfactory, and there is no agreement about its optimum treatment (19,21). In the last two decades, the large progress in the development of newer antiepileptic drugs has allowed us to have a vast choice of treatment options to control the spasms, although they often fail to do so. In our clinic, we observed that response to hormonal therapy was not as good as described in the western literature. Thus, the effect of other possible therapies on WS needs to be explored. 

Studies have reported that magnesium has antiepileptic and neuroprotective effects by antagonizing excitation through the NMDA receptor (14). Studies on the antiepileptic activity of magnesium have reported that in patients with epilepsy, serum magnesium levels are often low, and magnesium supplementation can be used as an adjunct to antiepileptic drugs (6-18). Therefore, we conducted this open-labeled randomized controlled trial to examine the effect of magnesium supplementation on children with WS. 

We carried out the present study with a prospective enrolment of subjects with a uniform treatment protocol and careful follow-up. A minimum follow-up period of 3 months was considered, and the outcome in the form of clinical response/seizure control, EEG improvement, and the neurodevelopmental outcome was studied in those who completed the follow-up. Uniform criteria were used for diagnosis, definitions, and outcome variables. Standard psychometric tests, like DST and VSMS, were used to assess the neurodevelopment status of the patients. The study comprised of a relatively larger number of patients (n=48) compared to other studies (38 children in Zou et al.’s study (2010) and 22 children in Carlen et al.’s study (2012)) and various case reports (16,18). We examined all demographic, clinical, and investigative factors that could possibly affect the response of the treatment of the children with WS.

In the present study, the intramuscular route was used in the first 3 days for magnesium supplementation followed by oral supplementation for 3 months. In a study by Zou et al. (2010), the intravenous route was used by giving higher doses of magnesium for 3 consecutive weeks. Oral supplementation used in this study is much safer and easier to administer than supplementation through the intravenous route used by Zou et al. (2010) (18). 

The clinical profile of WS patients in this study was almost similar to that in other Indian studies. The mean age of onset of spasms was 7.25 ± 6.69 months. Perinatal insults, like hypoxic-ischaemic encephalopathy (HIE) and neonatal illness, were associated in the most of our patients. The major findings of our study regarding neuroimaging were diffuse cerebral atrophy and encephalomalacic changes due to sequelae to perinatal HIE in 77% of the cases. We also observed hypsarrhythmia in a relatively small number of the patients. Similar results were found by Kalra et al. (2002) and Singhi and Ray (2008) (3,20). The present study results also indicated that serum magnesium levels were low in 17 of the patients (35.41%), and the mean serum magnesium level was 2.07 ± 0.5 mg/dl. 

After 3 months of magnesium supplementation, no significant difference was found between both groups regarding seizure control (p=0.693) and neurodevelopmental outcome (P=0.094). However, EEG normalization (p=0.003) was significantly more in the intervention group compared to the control group. Also, within the intervention group, seizure control was better in patients with normal initial serum magnesium levels than in those with low serum magnesium levels (p=0.003). We also found that patients with poor clinical response had relatively lower initial serum magnesium levels (1.73±0.44 mg/dl), which is in line with studies by Sinert et al. (2007), Gupta et al. (1994), Oladipo et al. (2007), McDonald et al. (1990), and Alan and Sander (2012) (6,7,8,14,15). Hypomagnesaemia may cause or exacerbate episodes of seizures as it has been seen that magnesium is a potential modulator of seizure activity that antagonizes excitation through the NMDA receptor and has antiepileptic and neuroprotective effects.

We observed that within the intervention group, seizures control was significantly better in patients with normal initial serum magnesium levels than in those with low initial serum magnesium levels. However, this difference was not significant in the non-intervention group, even with comparable serum magnesium levels. This means that magnesium supplementation increases serum magnesium levels and causes a better clinical response with normal serum magnesium levels. However, patients with low serum magnesium levels might not have a good clinical response. This can be explained by the fact that serum magnesium in patients with lower levels cannot increase to a level where it can effectively act as an anticonvulsant with supplementation at the dose of present study. Magnesium in our study was supplemented at a low dose and used for a shorter duration, mainly by the oral route. Thus, further studies need to be conducted at higher doses of magnesium and by following patients for a longer duration and changing the route of administration to increase bioavailability. Studies by Carlen et al. (2012), Van der Bergh et al. (2011), Zou et al. (2010), and Wan et al. (2019) also support that supplementing oral/parenteral magnesium causes improvement in seizures control (16-18, 22). 

Zou et al. (2010) (18) conducted a randomized controlled clinical trial and compared the effect of magnesium supplementation in children with WS for seizure control and neurodevelopmental outcome, as well as for EEG and adverse events. They gave higher doses of magnesium (250 mg/day) intravenously for 3 weeks and assessed efficacy after 6 months of follow-up. However, we provided a low dose of magnesium, which was almost equal to the recommended daily dose. We also used the intramuscular route for the first 3 days, followed by oral supplementation for 3 months. They found that the personal-social neurodevelopmental outcome significantly improved from baseline in the group receiving combination treatment (p<0.05). In this study on infants with WS, the proportions of seizure-free patients from 4 to 24 weeks were significantly greater in the intervention group than in the non-intervention group (91.7% vs. 70%). However, in our study, there was no significant difference in seizure control and the neurodevelopmental outcome between both groups. However, there was significant EEG improvement in the intervention group than in the non-intervention group. The difference in both studies might be since they used higher doses of magnesium by the intravenous route, which might have been more effective due to increased bioavailability, and that they followed the patients for a longer duration of 6 months. We used the oral route for magnesium supplementation after the first 3 days of intramuscular injections, which is much safer than continuous intravenous injections, and we did not observe any serious side effects due to magnesium supplementation. However, we used lower doses of magnesium and followed the patients for a shorter duration of 3 months. This might be the reason for the lack of significant clinical response in the intervention group and the erratic bioavailability of oral preparation.

In our study, there was no significant improvement in DQ/SQ levels in the intervention group, which contradicts the study by Zou et al. (2010) (18). They found significant improvement in DQ/SQ levels (p<0.05) in the intervention group. Reasons may be that most of our patients ( 90%) belonged to the symptomatic group and the commonest etiology was HIE, which is an irreversible process. Another reason might be that we followed our patients for a shorter duration of 3 months. Although the difference in the DQ/SQ level was nearly significant (p= 0.09), the overall long-term developmental outcome was not good at all, even after the successful treatment of infantile spasms. This is in line with Mackay et al.’s study (2004) (19) but is in contrast with Zou et al.’s study (2010) (18). 

**In conclusion,** although no significant improvement was found in seizure control and the neurodevelopmental outcome after magnesium supplementation, significant EEG improvement was observed. Also, within the intervention group, clinical response was better in patients with normal initial serum magnesium levels than in others. Thus, magnesium supplementation may be helpful in children with WS. However, more studies are required with a higher dose of magnesium supplementation, a larger sample size, and longer follow-up. 

## Authors’ Contribution

VK, A, SY, and KK conceived the hypothesis and concept. VK collected data. A and SY did laboratory work, KK did data analysis, and VK, KK, and RK wrote the manuscript. All the authors approved the final version of the manuscript.

## Conflicts of interest

None 

## References

[1] Lux A L, Osborne J P (2004). A proposal for case definitions and outcome measures in studies of infantile spasms and west syndrome: consensus statement of the west Delphi group. Epilepsia.

[2] Fukuyama Y (2001). A special note on terminology of West syndrome and infantile spasms. Brain Dev..

[3] Kalra V (2002). Gulati S. West syndrome and other infantile epileptic encephalopathies- Indian hospital experience. Brain Dev..

[4] Hancock EC, Osborne JP, Edwards SW (2008). Treatment of infantile spasms. Cochrane Database syst. Rev..

[5] Tsao CY (2009). Current trends in the treatment of infantile spasms. Neuropsychiatr Dis Treat..

[6] Sinert R, Zehtabchi S, Desai S, Peacock P, Altura BT, Altura BM (2007). Serum ionized magnesium and calcium levels in adult patients with seizures. Scand J Clin Lab Invest..

[7] Gupta SK, Manhas AS, Gupta VK, Bhatt R (1994). Serum magnesium levels in idiopathic epilepsy. J Assoc Physicians India..

[8] Oladipo OO, Lesi FE, Ezeaka VC (2007). Plasma magnesium and calcium levels in children with epilepsy in lagos. Niger Postgrad Med J..

[9] Miyamato Y, Yamamoto H, Murakami H, Kamiyama N, Fukuda M (2004). Studies on cerebrospinal fluid ionized calcium and magnesium concentrations in conclusive children. Pediatr Int..

[10] Prebble JJ (1995). Primary infantile hypomagnesenia: report of two cases. Paediatr Child Health..

[11] Carney PR, Dharnidharka VR (2005). Isolated idiopathic hypomagnesemia presenting as aphasia and seizures. Pediatr Neurol..

[12] Leaver DD, Parkinson GB, Schneider KM (1987). Neurological consequences of magnesium deficiency: correlations with epilepsy. Clin Exp Pharmacol Physiol..

[13] Lynch BJ, Rust RS (1994). Natural history and outcome of neonatal hypocalcemic and hypomagnesemic seizures. Pediatr Neurol..

[14] McDonald JW, Silverstein FS, Johnston MW (1990). Magnesium reduces N-methyl-D-aspartate (NMDA)-mediated brain injury in perinatal rats. Neurosci Lett..

[15] Yuen AW, Sander JW (2012). Can magnesium supplementation reduce seizures in people with epilepsy? A hypothesis. Epilepsy Res..

[16] Carlen PL, Abdelmalik PA, Politzer N (2012). Magnesium as an effective adjunct therapy for drug resistant seizures. Clin J Neurol Sci..

[17] Van den Bergh WM, Visser NA, Braun KP, Leijten FS, van Nieuwenhuizen O, Wokke JH (2011). Magnesium treatment for patients with refractory status epilepticus due to POLG1- mutations. J Neurol..

[18] Zou LP, Wang X, Dong CH, Chen CH, Zhao W, Zhao RY (2010). Three week combination treatment with ACTH+ magnesium sulfate versus ACTH monotherapy for infantile spasms: a 24-weeks, randomized, open-label, follow up study in China. Clin Ther..

[19] Mackay M (2004). Practice Parameter: Medical Treatment of Infantile Spasms: Report of the American Academy of Neurology and the Child Neurology Society. Neurology..

[20] Singhi P, Ray M (2005). Profile of West syndrome in North Indian children. Brain Dev..

[21] Nelson G R (2015). Management of infantile spasms. Transl Pediatr..

[22] Wan L, Yang G, Zou LP, Wang J, Shi XY, Ren WH, Lu Q (2019). Factors in first-time adrenocorticotropic hormone therapy and their influence on spasm control time in infantile spasms: a Cox proportional-hazards regression model analysis. CJCP.

